# Practical Hygiene1Read at the annual meeting of the Medical Society of the County of Chautauqua, July 10, 1900.

**Published:** 1900-09

**Authors:** Henry R. Hopkins

**Affiliations:** Buffalo, N. Y., Professor of hygiene, University of Buffalo; chairman committee on hygiene, Medical Society of the State of New York, 433 Franklin Street


### BUFFALO MEDICAL JOURNAL.

VOL. XL.—LVI. SEPTEMBER, 1900. NO. 2.

### Original Communications.

### PRACTICAL HYGIENE.[1]

BY HENRY R. HOPKINS, M. D., Buffalo, N. Y.,

Professor of hygiene, University of Buffalo; chairman committee on hygiene, Medical Society of the State of New York.

THE compliment and pleasure of an invitation to address your honorable body is more highly appreciated and becomes more of a red-letter event to me from the fact that it was my good fortune to spend the years of my boyhood in this most favored county. Wander where he may the sky is never so blue, the foliage never so fresh, the smile of friendship never so charming as in his native land, and Chautauqua is the land of my birth.

My purpose is to speak to you upon some of the points in hygiene that it would seem should be of special interest to those who have the onerous duty and responsibility of medical advisers to a rural people. The art of hygiene is most uniform in its aims and objects, but the demands upon that art of our people who live in our cities and towns, and of our people of rural life differ as the details of their several environments.

You will perhaps indulge me in a single observation of a general character as to the status of the work of hygiene at this time. Look where he may the student of hygiene finds cause for encouragement; the laboratory, the journal, the text-book, the field of battle, the camp, the battleship, the transport, the school, the theatre, the auditorium, the urban home and the rural home, the operating-room and the lying-in chamber, the sick-room everywhere gives evidence of the benign life-saving and hope-giving work of an improved, a more precise, a more efficient hygiene. On every hand opinion and uncertainty are giving way to conviction and precise methods, and on every hand the results in preventing disease and lessening mortality

1. Read at the annual meeting of the Medical Society of the County of Chautauqua, July 10, 1900.

are most encouraging and stimulating to yet greater effort and higher aims.

In the State of Michigan the records show that between the years 1887 and 1894, there were 738 outbreaks of scarlet fever and diphtheria, with an average of more than thirteen cases of illness to each outbreak. Since 1894 with improved methods of notification, isolation and disinfection, there have been 412 outbreaks with an average of cases to each outbreak of less than 3. In the City of Buffalo during the four years ending with 1884, there were 1696 deaths from scarlet fever and diphtheria; while for the four years ending with 1898 there were 838 deaths from these diseases. In this period from 1884 to 1898, the population of Buffalo has doubled and the mortality from scarlet fever and diphtheria has been lessened by one-half. During the American-Spanish war there was not a single death reported from contagious or infectious disease from our navy, and that notwithstanding the fact that the men were exposed to the burning heat of summer in the torrid zone.

Let us bow down to the faithful men whose patient, modest and scientific investigations have laid so sure a foundation for that most beneficent of all the handmaids of human progress, the science and the art of hygiene.

I wish now to invite your attention to some observations upon the hygienic management of three of our more pervalent and important diseases, it being my purpose to emphasise with greater particularity those points in the management of such diseases as relate to the responsibility of medical practitioners among rural people. For this purpose we will consider tuberculosis, typhoid fever, and scarlet fever.

### TUBERCULOSIS.

Tuberculosis concerns us from its universal prevalence and enormous death-rate, and it never seems amiss to repeat that which we so well know, that this disease causes about one-seventh of all our mortality; that the annual mortality in the State of New York is 13,000, and that of this vast caravan, which with steady, unbroken and unending tramp day by day, month by month, and year by year, without hope or expectation, march to untimely graves, our rural population furnish their full quota.

The discovery of the bacillus of tuberculosis seems destined to play an important part in shaping our notions of the therapeutics of tuberculosis, as it has already done in transforming our ideas as to its pathology and etiology. We do but state our common beliefs and fears when we remind you that if not now, certainly in the near past the prognosis of tuberculosis was hopeless. In this statement I do not forget the rare cases—the exceptions to the usual experience—but have in mind the vast mortality—the common dread, the popular hopelessness.

We have been accustomed to say when driven to it: Sir, I fear you have consumption; and we are accustomed to hear from the ashen lips of the condemned: Doctor, how long can I live?

Gentlemen, since the angelic message at Bethlehem, telling of the birth of the Christ, there has been no more hopeful gospel to mankind than that of Koch's bacillus, whereby the diagnosis of consumption may be made early in the disease; made months and years earlier than we are used to make it, or dare to speak the word; made at a time when the disease is curable; and curable in the large majority of cases—curable by the simple use of God's great remedies—hope, pure air, sunlight and plain food. It is our great privilege to hear these good tidings of great joy, and to bear an honorable part in this hope-giving life-saving campaign.

These observations apply to us as to all members of the medical profession, but there are others to which I would urge your attention, that more especially concern ourselves. Experience, observation and experiment unite in the demonstration that tuberculosis may be acquired by the lower animals, those more frequently found upon the farm, whose flesh and other products are used for human food. The spread of consumption from man to the domestic animals and from these animals back to man, indicates that the responsibility of the medical man of rural practice in regard to tuberculosis is three-fold; he must so manage cases of this disease that the sufferer may have his chance for recovery, so control the infection that it may not spread to the family or friends, and furthermore, that the infection may not spread to any member of that larger family the fowls and domestic animals of the farm. Fortunately, to do all this imposes neither hardship nor complicated and exacting regimen. The suptum must be recognised as the infecting medium; must be received in a suitable receptacle, and must be promptly destroyed. When it becomes generally known that the dry sputum of the consumptive is as dangerous as the falling scab of small-pox, the intelligence of the people may be trusted to find suitable means for their protection and its destruction.

Positive convictions upon the part of the members of our profession as to the communicability of tuberculosis and as to the various ways whereby such communication is effected, is the first step yet to be taken in this most important life-saving crusade.

### TYPHOID FEVER.

The case against typhoid fever may properly be introduced with the important and startling observation that typhoid fever in this State is a rural disease; that the bacillus typhosus, whose habitat and ravages were formerly associated with the dirty and crowded camp, the filthy and unventilated tenement house and transport ship is now chiefly at home in the smaller villages and farms of that part of our country, the rural districts, where a large part of our whole people dwell, where we are accustomed to think of the conditions of living as absolutely hygienic.

The health authorities of the State of New York, in order to the more intelligent study of the public health, divide the State into eight sanitary districts. These several districts present a wide variety of conditions known to be important factors in determining the morbility and mortality of the people.

The Maritime or first sanitary district has a population of 1,535 to the square mile, and of this population 90 per cent. are urban. Of all the districts this is the more densely populated and has the higher percentage of urban population.

The other extreme of condition is found in the Adirondack or third district, where the density is reduced to 26 to the square mile, and of these only 7 per cent. are urban. Between these extremes the conditions of the other districts are found and present a more uniform environment.

The experience as to the mortality from typhoid fever is uniform in this respect, that of a 1,000 deaths from all causes the number from typhoid fever is constantly higher in the districts having a larger proportion of rural population. The picture of the last year is practically good for the ten years last past, and that gives the Maritime district out of each 1,000 deaths 8 per cent. of typhoid fever, and the Hudson Valley district, 20 per cent. from the same cause.

The latter district has for many years had the highest typhoid mortality in the State—18, 17, 15, 16 per cent. are the proportions of some of the other districts, 16 per cent. being that of our own, known as the Ontario and Western district. You will bear in mind this is not a vagary of a single season, the effect of some temporary cause, but the average experience of many years and seems to indicate that the country well is frequently too near to the privy. That the bacillus typhosus has found a congenial home in "the old oaken bucket, the iron-bound bucket, the moss-covered bucket, which hangs in the well."

The experience of the State seems to be likely to follow that of twenty years or more ago of the City of Buffalo. Typhoid was epidemic throughout the entire year, and intelligent opinion began to indite the city well. This was before the days of bacteriology, but was not before the days of the chemical analysis of drinking water, and such analysis showed that our wells were rich in ammonia of albuminoid origin; that that ammonia was of human origin; that it was the product of human excrement; that our wells and our deepest wells where the dependent places into which the soil saturated with the overflow from the privies and sewer leaks was being drained, and our practical medical men, guided by their artistic instincts declared: that this is a sufficient demonstration—the drinking of this polluted water causes typhoid fever and these wells must be closed in the interests of public health! The wells were closed, our people were forced to use the pure water of the Niagara river, and our death-rate from typhoid fell most satisfactorily. Years afterward the discovery of the bacillus typhosus gave the means of making a scientific demonstration of what was before an artistic conclusion, and what was possibly more important, gave an important testimony to the nature of the operation of the mind that the mind can and does reach a true conclusion even in the absence of some important links in the evidence.

And what are the conclusions that we are to draw from the astonishing fact that in the State of New York typhoid fever has been for years and is a rural disease. Starting from the thorough conviction of clinicians and laboratory workers that typhoid fever is a water-borne disease, we will be correct in our finding that the farm well is the main source of danger from this disease, and that the farm well must be protected from ground water carrying in suspension or in solution the products of human excrement. Again, that rural practitioners of medicine must be more careful in their diagnosis; that they must treat fewer cases of simple fever, of bilious fever, of malarial fever, of autumnal fever, of biliousness, of indigestion with fever, because it is worth while to be right and these diseases are rare and infrequently seen. Again, rural physicians must see more cases of typhoid fever, because to treat these cases without seeing their true infectious character is to neglect to take those simple and inexpensive hygienic precautions to neglect to sterilise the typhoid dejecta, to fail to prevent the infection from spreading, and is to become responsible for the poisoning of the well, the poisoning of the spring, the poisoning of the stream at its source; and we must not forget that if the spring, the head waters of the stream are poisoned with the bacillus typhosus, whoever drinks of that spring or of that water course, miles and miles below, drinks to his death.

Again, it is worth while to remember that the farm is the source of the food supply, and as we have before seen that it is inexpedient that the farm should send to market tuberculous poultry, tuberculous meat, tuberculous milk, so likewise is it inexpedient that the farm should send to market vegetables, fruits or other farm products which have been washed in water infected with bacillus typhosus. And, lastly, the farm is the home of the dairy, the source of one of the chief foods of all, and the principal food of the young and susceptible, cow's milk. Experience amply demonstrates that it is important that milk cans be washed and rinsed in clean water, and fair-play would seem to demand that if we must have our milk watered, that the doctor should at least see to it that the diluent be free from the infection of typhoid.

I must close this part of my subject, but will leave in your minds Osler's vigorous etching: "This is God's country, with man's back-yard and the devil's cesspool."

### SCARLET FEVER.

I have selected this disease as the topic of final remark for the double reason that it would seem that something should be said of our hygienic treatment of it, and that we have a special responsibility from our field of work. It has previously been observed, as a cause for congratulation, that in many particulars the work of the bacteriologist has given precision to the knowledge and work of the hygienist, notably so in our knowledge of the bacillus of consumption and the bacillus of typhoid. Precise knowledge of how these infections leave the body, how they spread and communicate their different diseases after leaving the body has given us an accurate method in the management of such cases. We know that it is not essential to quarantine and isolate cases of consumption and typhoid fever. We know that it is only essential to sterilise the sputum of the consumptive, to sterilise the dejecta of the typhoid, and that this being done that the sufferer may remain at home or in the general ward of the hospital without danger to others. Regarding scarlet fever we are still obliged to confess that its specific principle has not yet been detected and therefore that our knowledge of it is wanting in scientific foundation, but it seems to me that our practice in these cases is scarcely up to the beliefs of those of us who have given the disease the more careful study.

Speaking from study and from observation it is my belief that scarlet fever is during its first days, before the beginning of desquamation, but feebly contagious and not at all infectious; that it becomes infectious with the peeling and that the infection leaves the body chiefly or only in this manner. My practice has for many years been governed by these beliefs and ample experience seems to justify the method.

Scarlet fever and measles are quite closely associated in the minds of both profession and laity and in hygienic management and curative treatment we are wont to think of them along similar lines; while the fact is they have nothing so conspicuous as their contrasts and may be treated with widely differing hygienic methods. Scarlet fever is infectious; measles is not. Measles is highly contagious, scarlet fever feebly so, if at all. The invasion of scarlet fever is abrupt, explosive; that of measles is slow, frequently lasting days or a week. The period of incubation in scarlet fever is short—three to five days; that of measles long—ten to fifteen days. Isolation to be of any use should be enforced in measles from the exposure, or at the end of the period of incubation, while in scarlet fever it is efficacious at any time during the early days of the eruption. With the coming of the eruption in measles the communicability of the disease seems to be over; in scarlet fever the fading of the eruption shows that the period of communicability is about to begin.

These notions regarding scarlet fever result in hygienic methods, to which I would briefly invite your attention. The principle of isolation is the more important principle of preventive medicine, and in no disease is isolation more adapted than in this. The art of hygiene is so advanced that our standard should be, that save in cases of general infection, there should not be the second case of scarlet fever in any family where the case comes under observation during the first week of the disease. Over and again, the situation is repeated. There are half a dozen children in the family, they all play in the same room, they all sleep in the same room and frequently two or three of them sleep in the same bed. One is taken ill in the night; you see the case the next day, and find it well out with the rash; you make your diagnosis and suggest isolation and the mother says: how useless, they have all slept in the same chamber, and two in the same bed. Too frequently we give way to the objection, fail to isolate the case and have six cases where there need be only one. Isolation should be practised in all cases of scarlet fever and may be depended upon to prevent the spread of the disease at any time before the stage of desquamation. Furthermore, hygienic treatment, preventive measures, may properly be instituted from the first. We do not know the microbe of scarlet fever, but we have every reason to believe that there is one and that it is susceptible to the attack of disinfectants and germicides, as are the microbes of the other infections, and from the first day of treatment measures should be instituted to disinfect the skin, and more particularly the portions of the surface covered by hair.

The stage of desquamation comes when it will, at variable periods, and to get ready for this process is the most essential part of the hygienists duty. In this part of our work, we may be guided by what we know and by what we believe. We know the infection leaves the body in the bran-like scales of epidermis, and we know that the lighter these scales are the further the air currents will be able to carry them. Knowing this we begin our effort to limit the sphere of infection of a given case, by rubbing the body several times each day with lard or some other oily substance for the single purpose of loading the scales that are to be thrown off—that they may not fly far. Fortunately, these inunctions are antipyretic and may be repeated with such frequency as the thermometer indicates, but should be used several times daily as a preventive. The hairy portions of the surface should in like manner have a daily bath in some liquid disinfectant, alcohol being reliable and having properties likely to make it popular with nurse and patient.

In this we are guided by knowledge; our beliefs will direct that the lard or other oily substance contain some disinfecting principle and fortunately nature has armed us with a large class of bodies, the essential oils, that are powerful germicides and therefore efficient disinfectants. These oils are reliable in varying proportions from 1 to 100 to 1 to 35,000. Oil of mustard having the higher proportion has great reputation as a disinfectant for the hands of the operating surgeon and should be efficient in quantities that would be free from its well-known effects as an irritant. Oil of eucalyptus is not so powerful a germicide, but is less likely to prove irritating and from long use I can advise it in strengths varying from 1 to 10 per cent., regard being had for the delicacy or density of the skin. The proper proportion may be added to a cup of melted lard, well stirred and allowed to cool, and you have a potent remedy representing knowledge and belief, the science and the art of medicine. I hold that it is easily within the power of the physician so to conduct his cases of scarlet fever and diphtheria that in these diseases the room shall never become infected; that the room may be entered at any time during the course of the disease by the physician or parent without danger of spreading the disease, and I am aware that during the state of desquamation of scarlet fever this theory is the most seriously tested. In order that the room may not become infected, care must be exercised in its selection and eternal vigilance in management during the illness. Fresh air and cleanliness are the words which point the directions for our efforts and you would say that these are simple matters easy of accomplishment.

To wipe the bare floor daily with a cloth moistened in a suitable disinfectant solution, and—1 to 1,000 of corrosive sublimate answers well for this purpose—should be the minimum of care throughout the entire course, but when the peeling is in process this should be done more frequently. To provide fresh air—in this as in all other infections the great and unequaled disinfectant—is at once your most important, easiest and hardest duty. Why it is important, or how it may be accomplished, we will not spend time to discuss, but will turn our attention to the difficulties. First in order and importance should be named, the lack of conviction on the part of the physician, likewise his persistent belief that great care must be exercised in keeping the patient warm in all cases of exanthemas, likewise his still greater solicitude that there must be no draft, or chance for cooling the surface in any disease having a tendency to kidney complication—the well-known danger from nephritis in scarlet fever is the bug-bear of the disease—and the resulting close and foul rooms, are, in my judgment, the chief causes of this complication.

Scarlet fever is a toxic disease—the toxin is in the blood and in every vascular tissue. Scarlatinal nephritis is a toxic inflammation, and it is prevented or minimised by antitoxic management, and there is no antitoxin like the antitoxin of pure air, regularly adminstered day and night, summer and winter, throughout the entire course of the disease. After this manner was I taught by my preceptor, a master of the art of medicine, the late Dr. Gorham F. Pratt, and have so practised for thirty-five years, and during this time have not in private or hospital practice had a fatal or alarming case of post-scarlatinal nephritis. In my judgment, emancipation from the prevailing fear of the malign effects of fresh air in scarlet fever is necessary before a physician can exhibit the intelligence of the art of preventive medicine, as related to this disease, and when that emancipation is fully come, the physician's troubles begin, for he then finds himself at war—a pioneer conscience on the one hand and professional and popular prejudice on the other.

The second order of obstacles to the suitable airing of the sick room in this disease is the ignorant and invincible prejudices of the friends of the patient. You may give your orders clearly and precisely, as good form always directs, and your orders may be received with a reasonable show of loyal obedience, but ten to one those orders will be fanatically disobeyed the moment you are out of sight. Infinite patience and perseverance is the only rule to success in this work. Can you bring chemistry to your aid to prevent the room from becoming infected? There can be no doubt of your ability so to do. Pastiles containing materials which heat diffuses throughout the room and still retain germicidal power and yet leaves the atmosphere respirable are found in all the shops. Frequent cleansing of all parts of floors and fixtures where dust might rest with a vigorous disinfectant is, of course, in order and presents no serious difficulties.

The thought that should guide us in this work is that the infection of scarlet fever is a particulate body that is thrown off in the peeling; that it should be sterilised while yet upon the body, or not allowed to fly beyond the sick bed.

Finally, as to the particular duty of the rural practitioner in these cases. This consideration takes us back to a previous position where we saw that the farm is the source of food supply to our less fortunate urban population; and we have simply to remember that recent observation amply demonstrates that milk is a most sensitive and perfect culture medium for the microbe of scarlet fever; that at the present time infected milk seems to be the chief source by which the disease is communicated. The coincidence of the outbreak of scarlet fever and the milk route of a single dealer has been shown over and over again and of itself proves the relation of the disease to the milk supply; this becomes a demonstration, as Dr. Wende has reported in many cases, when search of the milkman's premises reveals a case of desquamating scarlet fever in his family, and brings out the point of where the responsibility of the medical adviser in the case comes in.

The country doctor is sometimes regarded as an unimportant or humble member of the body politic, that he does not need to know very much, that a slight knowledge of medicine and a large ability to resist hunger, and loss of sleep, cold and heat, make up the essentials of his kit; but we have seen that the country doctor stands upon guard at a most important and exposed part of the camp, and that to guard the lower animals from tuberculosis, to protect the wells and streams from the bacillus typhosus, to preserve the milk supply from the infection of scarlet fever is a duty and a service to the public health of the highest magnitude; and it hardly needs to be said in view of the prejudices, the isolation, the lack of sympathy against which he is obliged to work that his professional kit needs to be well stored.

To conclude I cannot do better than repeat the noble words of your honored presiding officer, "salus populi suprema lex" and would add the simple thought, that to be permitted even in a humble way to work in the cause of preventing disease, of protecting the health and the lives of the ignorant and helpless, is worthy the ambition of the most ambitious; that in this work sanitary laws and health ordinances, both state and local, must be conceived with full knowledge, and enacted in practical wisdom, but even then will fail miserably unless supported by sane public opinion and enforced by a medical profession, every member of which is generously equipped for great responsibilities. An efficient and intelligent medical profession is at once the beginning and the end of the equipment for the protection of the public health, and high standards of qualifications and state examinations to test the aspirant by these high standards, are but intelligent means to important ends.

433 Franklin Street.

